# Research on Influencing Factors of Food Choice and Food Consumption

**DOI:** 10.3390/foods12061306

**Published:** 2023-03-19

**Authors:** Maggie Geuens

**Affiliations:** Faculty of Economics and Business Administration, Ghent University, Tweekerkenstraat 2, 9000 Ghent, Belgium; maggie.geuens@ugent.be; Tel.: +32-9-264-3521

Nowadays, most consumers are well aware of what makes up a healthy and sustainable diet. A sizeable proportion of these consumers are also interested in eating healthy and sustainable foods. Nevertheless, many consumers struggle to resist the temptation of palatable food and eat more unhealthy and/or unsustainable food than they should, resulting in huge health and environmental problems worldwide. Turning the tide has proven to be difficult, mainly because food choice is driven by a multitude of factors. Four important determinants highlighted in prior research are as follows: (1) social factors such as parental style and household eating habits; (2) product-related factors including product attributes, packaging, and labels; (3) personal factors, for example, food knowledge and cooking skills; and (4) situational factors such as food availability, time pressure, and store design. This Special Issue aimed to add to this body of knowledge by reevaluating prior determinants and testing new determinants of the choice of (specific types of) food.

First of all, several contributions point to a strong effect of social factors. Koch et al. argue that disgust associated with sustainable food alternatives such as edible insects and lab meat may be overcome by public exposure therapy. That is, formal institutions, retailers and food producers, opinion leaders, and parents and caretakers should change social norms by increasing consumers’ exposure to these foods and thus normalize them. If no longer perceived as deviant, edible insects and lab meat may reduce consumers’ disgust response, thereby increasing purchase likelihood [1]. The importance of social norms is also illustrated for hemp food and fast food consumption [2,3]. Metcalf and colleagues found that consumers’ beliefs concerning what family, friends, peers, and doctors would think of them consuming hemp food (a healthy and sustainable crop but which was illegal until recently in the country of investigation) and beliefs concerning the similar behavior of others contributed most to consumers’ intentions to consume hemp food [2]. Similarly, Bîlbîie et al. reported social norms (especially injunctive norms) as the strongest drivers of fast food consumption, while personal factors (such as attitudes, cooking skills) and situational factors (i.e., lack of time) have only a small effect [3]. Social factors thus unfortunately can also sustain or instigate undesirable behavior. The latter was observed by Rizzoli and colleagues when investigating the conversations and information spread on social media concerning food-borne diseases. Communities for pregnant women appear to regularly minimize or deny food risks and pay little to no attention to the most widespread food risks, calling for interventions of health institutions to promote correct food behavior [4].

Second, several contributions in this Special Issue point to new or revised conclusions concerning product-related factors. Three contributions investigated package-related elements. To reduce consumers’ intentions to eat meat, Choueiki et al. suggested using on-pack stickers with an anthropomorphic message stressing the negative consequences of meat production for the animals (such as pain and social or intellectual suffering). Anthropomorphism apparently makes it harder to deny that animals have minds, leading consumers to feel guilty about eating meat which in turn reduces their intentions to consume meat [5]. Tran et al. found that, also in a developing country (Vietnam), consumers are willing to pay a price premium for products whose packages include signals of food safety such as certifications, branding, and traceability labeling schemes. Not so much knowledge, but especially trust in the labels appeared to be important in this matter [6]. Investigating types of packaging, d’Astous and Labrecque found that a product package that is believed to be responsible (i.e., recyclable, reusable, compostable) triggers perceptions of the product being natural and healthy, which enhances consumers’ intention to buy the product [7]. Three manuscripts discuss product presentation styles. Meersseman and colleagues investigated the effect of showing pictures of food from which a bite was taken. Their conclusion was that marketers should avoid this practice as a picture of food with a bite elicited feelings of disgust which reduced consumers’ intention to purchase the food [8]. Mulier et al. investigated another popular trend in food pictures, implied motion, for a large variety of healthy and unhealthy foods. They could not replicate the positive effect of implied motion found in previous research, neither for unhealthy nor for healthy food [9]. Therefore, just as is the case for presenting food with a bite, presenting food in an implied motion style does not seem to be a recommendable practice. Adamczyk and Maison investigated the type of product benefits that are most convincing to make consumers switch to palm oil alternatives for chocolate spreads. Communicating environmental benefits was more effective than communicating health benefits in Spain, but in Poland health benefits worked better, at least for people who had an unhealthy dietary style [10]. Finally, Seubelt et al. compared the current diets of the German population (as evidenced by their food baskets) with food baskets based on a healthy diet, sustainable diets (vegetarian and organic), and differently priced baskets. Their results indicated that, for a large percentage of the population, price is the most determining factor, that is, they buy cheap food irrespective of its health or environmental impact, which implies a call for making healthy and sustainable food cheaper [11].

Five contributions in this Special Issue focus on a third category of determinants, i.e., individual factors. Modlinska and colleagues showed that there is a gap between accepting the idea of eating insect-based meat substitutes and accepting the actual consumption of such products. Food neophobia and disgust sensitivity play an important role in this [12]. Decreasing perceptions of disgust, for example, by means of public therapy as suggested by Koch et al. [1] may thus indeed pay off more than providing additional information. The importance of food familiarity was also stressed by Figueiredo et al. for rural provenance food. The choice for such rural food appears to be driven mostly by consumers’ familiarity with the rural territories, be it because of blood liaisons or because of frequently having visited the area. Next to familiarity, the extent to which consumers value the products’ sensorial aspects, the convenience, national provenance, and the impacts on rural development also matter [13]. Louro et al. studied the association between consumers’ taste sensitivity and food choice. Classifying consumers into three groups based on their taste threshold, they found that consumers that are less sensitive to most tastes (sweet, bitter, salty) showed higher intake of sweets, fast food, and animal-based food. Consumers that have a higher sensitivity for most tastes consume more low-fat dairy and salads, while consumers who are less sensitive to sour taste (which is the group with the higher BMI) show lower intake of fast-food and sweets, but higher intake of sausages and alcoholic beverages [14]. While a large group of consumers may enjoy meat, they may at the same time be concerned about the detrimental effects of meat on the environment, the animals, and human health, leading to ambivalent feelings towards meat consumption (cf., the meat paradox). Pauer et al. showed that meat-related ambivalence is relatively widespread in the general population, even though consumers show strong differences in this ambivalence. The authors also showed that making this ambivalence salient (which could, for example, be achieved by means of interventional messages or descriptive norms) motivates consumers to eat less meat. This can be explained by the anticipation of a relief of the ambivalent state engendered by changing their dietary pattern and an increase in seeking information that facilitates such dietary changes [15]. The fifth contribution on individual factors’ impact on food choice focuses on differences in terms of the cognitive load consumers experience during specific shopping trips, as well as in terms of how much they like to think (need for cognition) or how much they enjoy shopping. Carroll and colleagues showed that when consumers score high on the latter characteristics, they are more likely to choose food bundles (vs. single items), presumably because bundles demand less cognitive effort. Food bundles are interesting as they can be composed in such a way that they enhance healthy and sustainable food consumption [16].

A final paper looked into environmental factors. Mathiesen and colleagues investigated sound-proofing materials and playing music during lunch in a hospital for patients suffering from acquired brain injuries. They showed that improved acoustics and music have the potential to positively impact the lunch atmosphere and social interactions therein, leading to enhanced patient well-being, food intake, and nutritional state [17].

In sum, as the large body of prior literature and also this Special Issue illustrate, food consumption is a complex behavior that is driven by many factors. To ultimately arrive at sound recommendations to engender healthy and sustainable dietary patterns in the wide population, much more research is needed. Interdisciplinary research and field studies in which a myriad of factors can be investigated at the same time are especially called for.

## Data Availability

Not applicable.
